# Trait Performance Correlations across Life Stages under Environmental Stress Conditions in the Common Frog, *Rana temporaria*

**DOI:** 10.1371/journal.pone.0011680

**Published:** 2010-07-21

**Authors:** Frank Johansson, Baptiste Lederer, Martin I. Lind

**Affiliations:** Department of Ecology and Environmental Science, Umeå University, Umeå, Sweden; University of Turku, Finland

## Abstract

If an organism's juvenile and adult life stages inhabit different environments, certain traits may need to be independently adapted to each environment. In many organisms, a move to a different environment during ontogeny is accompanied by metamorphosis. In such organisms phenotypic induction early in ontogeny can affect later phenotypes. In laboratory experiments we first investigated correlations between body morphology and the locomotor performance traits expressed in different life stages of the common frog, *Rana temporaria*: swimming speed and acceleration in tadpoles; and jump-distance in froglets. We then tested for correlations between these performances across life stages. We also subjected tadpoles to unchanging or decreasing water levels to explore whether decreasing water levels might induce any carry-over effects. Body morphology and performance were correlated in tadpoles; morphology and performance were correlated in froglets: hence body shape and morphology affect performance within each life stage. However, performance was decoupled across life stages, as there was no correlation between performance in tadpoles and performance in froglets. While size did not influence tadpole performance, it was correlated with performance of the metamorphosed froglets. Experiencing decreasing water levels accelerated development time, which resulted in smaller tadpoles and froglets, i.e., a carry-over effect. Interestingly, decreasing water levels positively affected the performance of tadpoles, but negatively affected froglet performance. Our results suggest that performance does not necessarily have to be correlated between life stages. However, froglet performance is size dependent and carried over from the tadpole stage, suggesting that some important size-dependent characters cannot be decoupled via metamorphosis.

## Introduction

Organisms that live in different environments during ontogeny are faced with the constraint that it might be difficult to optimise responses, via adaptation, to the various selection pressures that operate in each habitat. Thus, trade-offs between physiological, morphological or behavioural traits might be constrained and so limit adaptation when ontogeny occurs across different environments [1]. It is therefore of interest to examine if and how phenotypic induction early in ontogeny affects later phenotypes. Such knowledge is important if we want to predict how changes in one environment affect phenotypes in another environment. For example, a rise in global temperature might affect the rate at which pools dry out, which in turn could affect the growth and development rates of aquatic insects and amphibians inhabiting these pools [2]. Frogs provide an excellent system in which to study how morphological and behavioural traits are related across life stages. This is because most frogs spend the first stage of their life in an aquatic environment, which selects for a different suite of traits than do the terrestrial or amphibious environments occupied by adults. While several studies have examined the relationship between morphological traits across life stages in frogs e.g. [3], [4], [5], [6], [7], few have examined how behavioural traits are coupled across the different stages. In the present study we focus on how morphology and locomotor behaviour is correlated across life stages. We simply ask whether larval stages (tadpoles) that perform well in terms of their locomotor behaviour in one environment, also perform well as juvenile adults (froglets) in another environment.

Locomotor traits are important for many aspect of a frog's life such as feeding, migration predator avoidance. Avoiding predation is important to amphibians, as it is one of the most common mortality factors during their larval and adult stages [8], [9]. It is not surprising, therefore, that amphibians exhibit adaptations with respect to their antipredator performance, both as larvae and as adults. However, performance often depends on morphology. For example, escape from predators is determined by the acceleration and speed of swimming tadpoles, which is dependent upon tail fin and muscle morphology [10], [11], [12]; hence, tail shape can explain some of the variation in tadpole survival in environments with predators [13]. In adults, one of the most important traits for affecting an escape from a predator is jumping, the jump length being related to morphological traits including leg length [14], [15], [16], and indirect evidence suggests that jumping ability does decrease predation risk [17], [18], [19]. Hence knowledge of morphology is important for a mechanistic understanding of antipredator performance across life stages.

Many amphibians, including frogs, encounter other environmental stress factors besides predation risk. One such stress factor is pool drying, to which tadpoles must respond by metamorphosing before all water is lost from the pool. The classic response to pool drying is to speed up development, either by having a fixed, short development time [20], [21] or by phenotypic plasticity [22], [23]. This usually comes at a cost in terms of smaller size at metamorphosis, which has negative effects on aspects of fitness in the adult stage [24], [25]. Some studies have also shown that the faster development, induced by pool drying, affects performance. For example, tadpoles exposed to simulated pool drying conditions developed a shorter and narrower tail fin [7], which morphologically impairs swimming speed [26]. In addition, froglets that metamorphosed in conditions simulating pool drying had a reduced jumping capacity [7]. However, these studies only measured performance within a single life-stage; how stress factors such as pool drying affect performance when measured on the same individual as tadpole and froglet, remains largely unknown. A valid question, therefore, is whether the phenotypic and genotypic correlations observed across life stages in a non-stressful environment, are the same as those seen in a stressful environment.

In this study we examine the relationship between locomotor performance of tadpoles and froglets of the Common Frog, *Rana temporaria*. We specifically address the following questions: 1) Do individuals that perform well as tadpoles also perform well as froglets? 2) How is the behavioural performance of tadpoles and froglets related to their morphology? 3) How does the relationship between locomotor performance and morphology within a life stage differ between unstressed tadpoles raised in a constant water environment, and stressed tadpoles raised in an environment with decreasing water levels simulating pool drying?

We answer these questions by first studying the relationship between morphology and performance within a life stage; we then examine the relationship between locomotor trait performances across life stages. We have no a priori prediction on how performance should be related across life stages (positive negative or no correlation), we simply ask how locomotor performance is correlated across life stages at the level of an individual. Our purpose is not to investigate the mechanistic basis of the performance, but rather the general locomotor performance. We therefore used an approach in which we reduce morphology and performance to single variable axes.

## Materials and Methods

For the studies on the performance and morphology of tadpoles and froglets, we collected eggs of the Common Frog, *R. temporaria*, which breeds in both permanent and temporary bodies of water, and is the most widespread frog species in Europe [27]. Eggs were collected from six island populations in the Gulf of Bothnia southeast of Umeå, northern Sweden (Ålgrundet: 63°41′N, 20°25′E; Fjärdgrund: 63°40′N, 20°20′E; Lillhaddingen 63°39′N, 20°23′E; Sävar-Tärnögern: 63°45′N, 20°36′E; Stora Fjäderägg: 63°48′N, 21°0′E; Storhaddingen: 63°40′N, 20°24′E). We sampled populations from six islands, not to compare populations, but to reduce any potential population or environmental effect on the variables we measured. The islands were visited on the 6th and 7th of May 2008, and from each island, samples of 20–50 eggs were taken from 10 clutches. If several pools were present on an island, clutches were collected and brought back to the laboratory from all pools with eggs. Because this species usually lays only one egg clump per season [28], each egg clump was assumed to have been laid by a separate female *R. temporaria*.

Clumps of eggs were kept in separate containers filled with conditioned non-chlorinated tap water, and stored in a constant-temperature room at 4°C until all eggs had been collected from the field. Conditioned water was produced by mixing three litres of dried *Betula pendula* leaves with non-chlorinated tap water in a 500 litre tank. Leaves were removed when the water was transferred to the experimental containers to eliminate any possibility of surplus food. To initiate egg development, the room temperature was set to a constant 22°C, and a day-night cycle of 18 and 6 hours, with the light switched on at 04:00 hours and off at 22:00 hours. This light regime corresponds to the natural cycle in Umeå, Sweden, at this time of the year. Light was provided from four fluorescent light tubes in the ceiling.

The experiment was started (Day 1) when the tadpoles had reached Gosner stage 23 [29], i.e. active swimming. Four individuals from each clutch (two for each hydroperiod treatment) were randomly chosen and placed in individual plastic containers (9.5 cm×9.5 cm×10 cm) filled with 750 ml of the conditioned water. Each container was coded and placed in the constant-temperature room, following a predetermined random distribution to reduce the effect of any environmental variation. In total 240 tadpoles were used in the experiment: six islands × ten females per island × two water level treatments × two replicate tadpoles per treatment combination.

Initially, tadpoles were fed daily with 15 mg (±0.1 mg) of food, consisting of a 1∶1 mixture of commercial fish flakes and rabbit food, finely ground using a pestle and mortar. During the course of the experiment the food levels were increased according to the following schedule: 30 mg from Day 9–12; 45 mg from Day 13–16; 60 mg from Day 17–20; and 75 mg from Day 21 until metamorphosis. Every fourth day, prior to feeding, the conditioned water was replaced with a fresh batch.

In order to examine the impact of an environmental stress on morphology and performance, we simulated pool drying for half of the tadpoles by decreasing the water level by 33% every fourth day. After Day 25, the water level was kept constant at a final volume of 66 ml. Tadpoles from the constant water level treatment were kept in a fixed water volume of 750 ml until metamorphosis.

To control for maternal effects through egg size [30] we measured 10–30 eggs from each clutch before hatching. Eggs were placed in a Petri dish and completely submerged in water in order to prevent the gelatinous capsule surrounding the eggs distorting the image. The eggs, together with a scale, were photographed using a vertically placed digital camera (Canon EOS 350D) fitted with a macro lens (Tamron SP AF 90mm, F/2.8 Di Macro 1∶1). Egg sizes were calculated using the image analysis software ImageJ v. 1.36b (http://rsb.info.nih.gov/ij/). Because maternal effects expressed through egg size did not significantly influence any of the other variables, egg size was excluded as a variable from all further analyses. For example egg size and jump length and eggs size and swimming performance showed no correlations (r^2^<0.03 and P>0.25 for both correlations).

### Tadpoles

**Figure 1:**
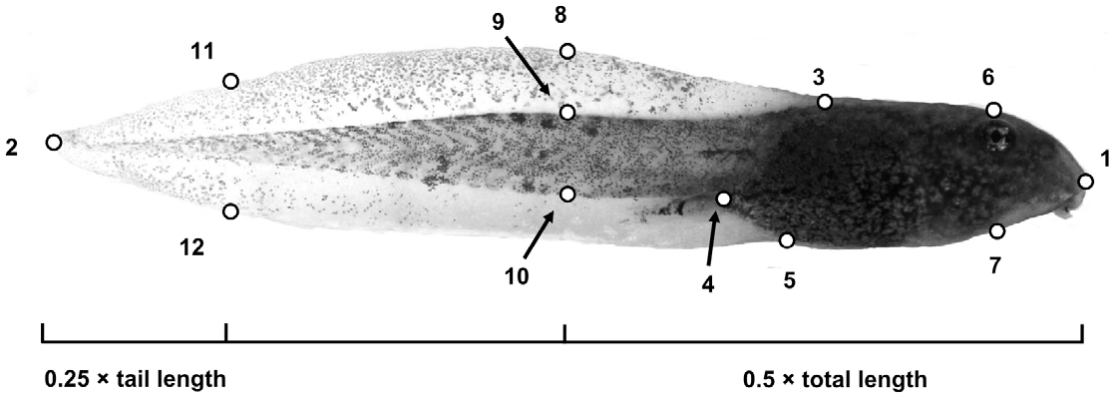
Tadpole showing the twelve landmarks used to analyse body-shape. The landmarks were selected to capture the overall body-shape on the basis of either morphologically or geometrically well-defined points. Landmarks were: 1: snout tip, 2: tail tip, 3: dorsal edge of tail fin attaching to head, 4: ventral edge of tail muscle attaching to head, 5: ventral edge of tail fin attaching to head, 6: head above eye centre, 90° dorsal of the central plane, 7: head below eye centre, 90° dorsal of the central plane, 8: dorsal tail fin edge at mid body length, 9: dorsal tail muscle edge at mid-body length, 10: ventral tail muscle edge at mid-body length, 11: dorsal tail fin edge at ¼ of tail length, 12: ventral tail fin edge at ¼ of tail length. Landmarks 6–12 were added with the aid of a grid layer placed over the image.

The morphological characteristic of tadpoles chosen for quantification was overall body-shape as defined by a number of landmarks that are commonly used in the analysis of shape and morphology of tadpoles, and which have been shown to be associated with performance and survival [7], [12], [26]. Body-shape was estimated at Gosner stage 37 [29], when the toes of the hind limb are separated (Fig. 1). Lateral photographs of the tadpoles were taken by placing the tadpole in a small glass chamber (55 mm×30 mm×10 mm) filled with conditioned water. To minimize the effect of the horizontal angle in which the tadpole placed its tail, all images were first straightened with the *straighten* plugin (http://rsbweb.nih.gov/ij/plugins/straighten.html) in ImageJ. The body-shapes of the tadpoles were captured by digitizing 12 landmarks (Fig. 1) on each image, using the geometric morphometric software TpsDig v1.40 [31]. After photography, the swimming performance of each tadpole was estimated in an aquarium (25 cm×52 cm) filled to a depth of 1.5 cm with conditioned water. One tadpole at a time was gently placed in the aquarium and allowed to settle for about 30 seconds. We then carefully approached the tadpole with a 20 cm long plastic stick and touched the water surface, lateral to the body of the tadpole, at a distance of 2–3 mm from the tadpole. This elicited an escape response, which was filmed from above with a digital video camera (Sony DCR-SR190E). Three swimming events that each lasted about 3 seconds were recorded for each individual. Each individual was then returned to its container in the controlled environment room. The video clips of swimming performance were analysed with the video analysis program Tracker 2.16 (http://www.cabrillo.edu/~dbrown/tracker/). We estimated acceleration and maximum speed of the tadpoles, and the highest value of these estimates from the three runs from each individual were used in later analyses. We used the maximum value rather than the mean value, because some of the swimming events seemed not to be maximal by the eye of a human observer.

At Gosner stage 42 (when the forelimbs emerge) we estimated development time, live wet weight, and growth rate. Growth rate was estimated by dividing each tadpole's weight by the number of development days. The tadpoles were then transferred to larger containers (19 cm×15.5 cm×8.5 cm) and covered by a net to prevent escapes. The containers contained 90 ml conditioned water and were tilted slightly so that one end provided a terrestrial habitat for the newly metamorphosed froglets.

### Froglets

**Figure 2:**
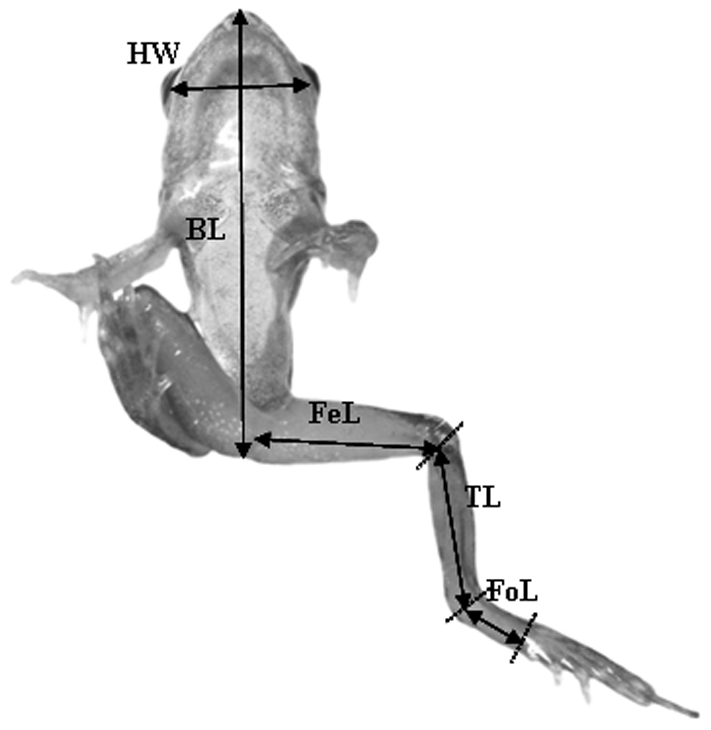
Juvenile frog showing the morphological traits that were measured. HW = head width, BL = body length, Fel = femur length, TL = tibia length, FoL = foot length.

At Gosner stage 46 the tail is resorbed and metamorphosis is completed. At this stage we assessed morphology and estimated the jump length of each froglet. Jump length was estimated by placing the frog on a piece of Styrofoam (60 cm×80 cm) and filming jumps with a video camera (Sony DCR-SR190E) placed above the arena. Jumping was initiated by approaching the frog with the end of a white plastic stick. Frogs always jumped before being touched, when the stick was at a distance of 1–2 cm. For each individual, three jumps were recorded and the distance jumped was estimated with the video analysis program Tracker 2.16. The longest of these three jumps provided the distance data used in the subsequent analysis. The longest jump was used rather than the mean value because, some jump events looked not as maximal attempts to a human observer. After the jumping experiment, body dimensions were measured by placing each froglet on a glass board with a scale. A camera was set up on a tripod beneath the glass board and the froglet was photographed from below. These images were used to measure five post-metamorphic morphological traits: head width, body length, femur length, tibia length and foot length (Fig. 2) using the program ImageJ v.1.37.

### Statistics

In all analyses, one individual per family was used as our replicate unit, with source population as a random factor. Although we initially used two individuals produced by each female (i.e. from a single family) as replicates in our experimental design, random mortalities, which mainly occurred during tail resorption between Gosner stages 42 and 46, resulted in the loss of uneven replication. Therefore, from the remaining paired replicates, we randomly picked one individual to provide a value for each family. A logistic regression confirmed that survival did not differ among populations (Z = −1.3, P = 0.2) or treatments (Z = 1.9, P = 0.06), although the treatment effect was only marginally non-significant and slightly more individuals died where water levels were kept constant (53%) compared to treatments with falling water levels (40%). The mortality figures are within the ranges reported in other studies where frogs have been raised from the egg stage until Gosner stages 45–46 with complete resorption of the tail [5], [6], [15].

The assumption of normality was analysed by visually plotting the estimate against the residuals: no strong associations between residuals and estimates were found. In cases where we used size-corrected methods, we always paid attention to the interaction between treatment and the covariate (size), since size-corrected methods can result in significant bias in the estimates [32]. Each treatment was analysed separately if this interaction was significant. Because the two variables, jump length and swimming performance, were used in several independent tests, we also provide Bonferroni corrected P-values for those cases where these variables were significant in our analyses. Principal components analyses (PCAs) were performed in SYSTAT version 12.02 [33]; all other analyses were run in R (http://www.r-project.org) using the NLME package.

### The effect of tadpole body-shape on swimming performance

Because tadpole swimming speed and acceleration at Gosner stage 37 were correlated (r = 0.57, P<0.001), we performed a PCA on these two variables to reduce dimensionality. The scores for the first principal component (PC1) was used as an estimate of ‘swimming performance’. This first axis from the PCA explained 78.5% of the variance. We therefore interpreted this axis as a good measure of overall swimming performance, and retained it for further analyses. A partial least squares (PLS) analysis was used to examine the relationship between swimming performance and the morphological characteristic ‘body-shape’ using the program TpsPls [34]. This program explores the relationship between body-shape and a set of other variables. In our case, the first component, describing swimming performance, was the only other variable. The landmarks captured from the software TpsDig [31] were entered to determine body-shape variables. The relationship between body-shape and swimming performance was tested by comparing the variable vector with the shape vector, using the permutation test available in TpsPls. To visually represent the relationship between body-shape and swimming performance, we plotted the shape projections (from the PLS) against swimming performance (the score from PC1). To visually represent the shape of the tadpoles in this plot, we generated grid plots to show shape of tadpoles in relation to the consensus (average body-shape of tadpoles). Grid plots were produced with the program TpsRegr [35], which explores the relationship between shape and one variable (in our case, performance). We also analysed the relationship between tadpole body-shape and swimming performance with a mixed model analysis of covariance (ANCOVA), using population as a random factor, water level treatment (constant and decreasing water) as a fixed factor, the PLS shape projection score as a covariate, and swimming performance as the response variable.

### Development time and weight at metamorphosis

The relationship between age and weight at Gosner stage 42 was analysed using a mixed model ANCOVA with population as a random factor, water level treatment (constant and decreasing water) as a fixed factor, age at Gosner stage 42 as a covariate, and weight as the response variable.

### Froglet performance

The effect of the morphology of froglets at Gosner stage 46 on jump length was analysed with mixed model analyses of variance (ANOVAs). We first determined PCA scores for the measurements of overall body morphology by performing a PCA using the variables head width, body length, femur length, tibia length and foot length. PC1 explained 72% of the variation and exhibited a positive correlation with all five variables, with correlation coefficients ranging between 0.73 and 0.93 (Table 1). We therefore interpret PC1 as a size variable. Since the scores from the first axis explained most of the variation it was used as a covariate in the model, with water level treatment as a fixed factor, population as a random factor, and jump length as the response variable. Since jump length could be affected by body size, we also analysed the effect of jump length independent of body size by using the score from PC2 in the PCA described above, and ran a similar ANOVA as for PC1. We used PC2 because the first axis in a PCA is usually associated with size and therefore PC2, which is orthogonal to PC1, should capture most of the size independent variation in morphology. PC2 explained 12% of the total variation and we interpret this axis as representing size independent aspects of morphology (Table 1). Foot length had a high positive correlation with PC2 score, and head width had a negative correlation with PC2 score, while the other three morphological variables had low component loadings (<0.12).

**Table 1:** Component loading for the variables included in the PCA relating to the morphology of froglets.

Variable	PC1	PC2	PC3	PC4	PC5
BODYL	0.86328	−0.04630	0.17184	−0.47133	0.03032
HEADW	0.73488	−0.62170	−0.26120	0.07087	0.01417
FEMURL	0.91414	0.11137	0.21787	0.25165	0.20286
TIBIAL	0.93708	0.05116	0.19450	0.15983	−0.23640
FOOTL	0.78839	0.44025	−0.42850	−0.03172	−0.00064

The percentage variance explained by each axis was 72.4 (PC1), 11.9 (PC2), 7.3 (PC3), 6.3 (PC4), 2.1 (PC5).

### Correlations between tadpole and froglet performance

The relationship between swimming performance of tadpoles at Gosner stage 37 and jump length of froglets at Gosner stage 46 was analysed with a mixed model using jump length as the dependent variable, swimming performance and froglet size as covariates, population as a random factor, and water level treatment as a fixed factor. We used the score for PC1 (performed on the five morphological variables described above) as our estimate of size for the froglets.

## Results

### The effect of tadpole body-shape on swimming performance

**Figure 3:**
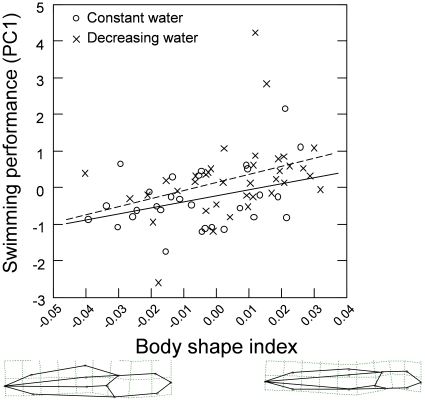
Relationship between shape index (shape projections from the TpsPls program) and swimming performance. Swimming performance is represented by a combination of acceleration and maximum swimming speed determined using a PCA. Grids on tadpole shapes show shape changes along the x-axis, and were generated with the thin-plate spline transformation in the program TpsRegr. The constant water level treatment is indicated by the solid line (y = 17.0x−0.2) and the decreasing water level treatment is indicated by the dashed line (y = 22.2x+0.1). Lower and upper confidence intervals for the regression coefficients are −0.0004 and 33.4, and 1.8 and 42.4 respectively.

The PLS-analysis showed that there was a positive correlation between body-shape and swimming performance (r = 0.41, P = 0.05; however, after Bonferroni correction this difference was no longer significant, P = 0.10). The relationship is presented graphically in Fig. 3: tadpoles that performed better in terms of swimming speed and acceleration had shallower bodies but higher tail muscles. The fastest individual's had a maximal acceleration and swimming speed of respectively 698 m/s^2^ and 59.8 m/s. After this initial analysis on shape and performance we used the shape projection scores in a mixed model ANOVA. This ANOVA yielded a non-significant interaction term between the shape of the tadpoles (obtained from the PLS) and the water level treatment (t_56_ = 0.42, P = 0.67). We therefore removed the interaction term from the model. Thereafter the model demonstrated a significant effect of morphological index on swimming performance (t_57_ = 2.82, P = 0.01). There was no significant effect of water level treatment on swimming performance, (t_55_ = 1.11, P = 0.27). A separate test of water level treatment with swimming performance removed from the model showed that tadpoles from the simulated pool drying treatment significantly differed in shape (t_1,59_ = 2.56, P = 0.013) from those from the permanent water, in that they had shallower bodies (Fig. 3). The size of tadpoles had no significant effect (F_1,61_ = 0.14, P = 0.72) on swimming performance and was therefore excluded from further analyses.

### Development time and weight at metamorphosis

**Figure 4:**
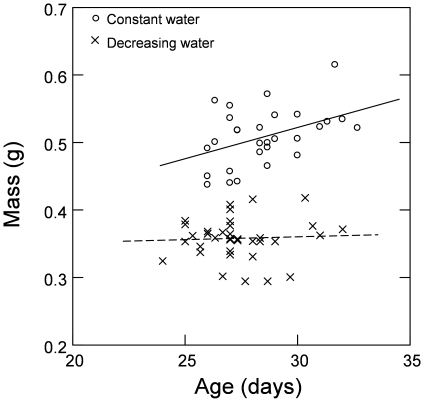
Relationship between tadpole age and mass at Gosner stage 42 under the two drying treatments. Regression statistics are (y = 0.010x+0.23, P = 0.016, r^2^ = 0.19) for the constant water level treatment (open circles, solid line); and (y = 0.001x+0.33, P = 0.73, r^2^ = 0.003) for the decreasing water level treatment (crosses, dashed line).

Tadpoles in the constant water level treatment exhibited a positive relationship between development time and weight, while no such relationship was found in tadpoles in the simulated pool drying treatment (Fig. 4). This finding was further supported by a significant interaction term in the mixed model ANCOVA (t_57_ = 2.04, P = 0.04). Separate linear regressions for each treatment also supported this pattern (see legend to Fig. 4).

### Froglet performance

**Figure 5:**
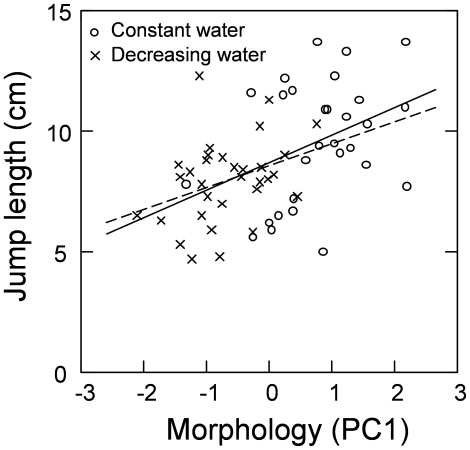
Relationship between body morphology and jump length. Body morphology is expressed as PC1 score. Froglets were subjected to the constant water level treatment (y = 1.2x+8.6; open circles, solid line) and decreasing water level treatment (y = 0.9x+8.6; crosses, dashed line). Lower and upper confidence intervals for the regression coefficients are −0.12 and 2.63, and 0.002 and 1.78 respectively.

PC1 scores (size dependent morphology) were also positively correlated with jump length, and a mixed model ANOVA showed that froglet morphology (PC1) had a significant effect on jump distance (t_53_ = 2.75, P = 0.008; P<0.01 after Bonferroni corrections), with higher scoring individuals jumping further (Fig. 5). In contrast, water level treatment did not affect jump length (t_53_ = 0.05, P = 0.96, Fig. 5). As there was no significant interaction between size (PC1) and treatment (t_52_ = 0.46, P = 0.65), the interaction term was dropped from the model. A separate test of water level treatment on size (PC1) showed that water level treatment significantly affected morphology (F_1,54_ = 10.07, P<0.001) in that froglets from constant water conditions were larger with respect to all measured morphological traits (Fig. 5). Frogs form the constant water treatment had an average body and femur length of 1.49 cm (±0.06 S.D.) and 0.62 cm (±0.05 S.D.) respectively, and frogs from decreasing water treatment had an average body and femur length of 1.37 cm (±0.08 S.D.) and 0.54 cm (±0.03 S.D.) respectively.

A second mixed model ANOVA run on the scores from PC2 (size independent morphology) showed that there was a significant interaction term between morphology and treatment (t_52_ = 2.30, P = 0.02). Since an interaction might bias our interpretation we ran the model separately for each treatment. For froglets raised with constant water levels, there was a tendency for PC2 score to have a significant effect on jump length (t_26_ = 1.90, P = 0.07), while for those raised with decreasing water levels there was no relationship between PC2 scores and jumping performance (t_26_ = 0.86, P = 0.39). However, both these comparisons were non-significant after Bonferroni corrections. Further, water level treatment had no significant effect on morphology, as described by PC2 score (F_1,54_ = 0.12, P = 0.72).

### Correlations between tadpole and froglet performance

**Figure 6:**
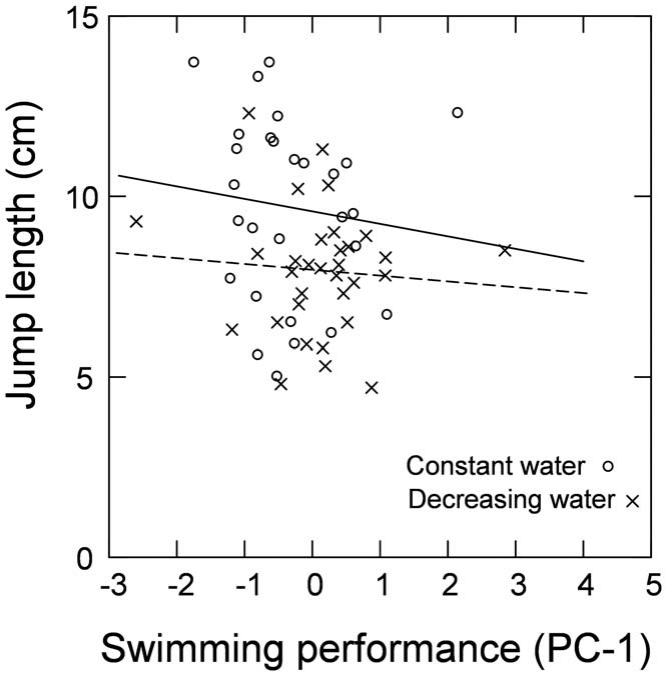
Relationship between swimming performance and jump length in individuals subjected to constant and decreasing water level treatments. The constant water level treatment is indicated by the solid line (y = −0.4x+9.5) and the decreasing water level treatment is indicated by the dashed line (y = −0.2x+8.0). Lower and upper confidence intervals for the regression coefficients are −1.57 and 0.88, and −0.88 and 0.56 respectively.

A mixed model ANCOVA showed that there was no significant effect of swimming performance on jump length (t_52_ = 0.62, P = 0.54), (Fig. 6). In contrast, the covariate (i.e. PC1 score) did have a significant effect on jump length (t_52_ = 2.53, P = 0.02; P<0.05 after Bonferroni correction), but water level treatment did not (t_52_ = 0.01, P = 0.99). Because the interaction between swimming performance and treatment was non-significant it was removed from the model (t_51_ = 0.05, P = 0.95). Furthermore, the lack of any significant interaction between PC1 score and treatment (P = 0.70), suggests that assumptions about variances in the covariate did not bias our data. We did not include the size of the tadpoles in this analysis since there was no relationship between tadpole size and swimming performance, as shown above. It should be noted, however, that tadpole size and PC1 score were positively correlated (r = 0.39, P = 0.001). In summary, this result suggests that while tadpole performance is not correlated with performance at the froglet stage if size is taken into account, size nevertheless does have a great effect, since large froglets can jump further than small froglets.

## Discussion

We found that the two locomotor performance traits studied, swimming and jumping, were not correlated across life stages. This suggests that performance was decoupled between life stages and that the environmental conditions affecting performance during early development do not necessarily equate with performance in later life stages. We also found that the tadpole performance traits, swimming speed and acceleration, were not affected by tadpole size, while the froglet performance trait, jump length, was positively correlated with froglet size. Hence it is possible that some of the decoupling of performance across life stages is driven by this size effect. In our study we cannot disentangle this size effect from the pure morphological performance effect that is driven by co-dependence from morphology. Thus, while metamorphosis decoupled the performance between the larval and juvenile adult stage, the performance of the metamorphosed individuals is functionally related to size, which is clearly determined during the tadpole stage, since tadpole size and froglet size were correlated. Therefore, undergoing metamorphosis does not represent a completely new start for froglets; instead the performance of the individual is still constrained by the burden of its developmental past, as discussed by Releya and Hoverman [36]. Our results contrast with those of Watkins [5], who did find a correlation between tadpole and froglet performance. However, it should be noted that in Watkins's [5] study this correlation disappeared after controlling for body size. Hence our interpretation of both his and our results, is that performances in the juvenile and froglet stages are decoupled, but that jumping ability is affected by the size of the froglet, which is carried over from the size of the tadpole. Finally, we are aware that the phenotypic correlations on which our study is based, are not necessarily adequate predictors of the underlying genetic correlations [37], but see [38]. But we would like to emphasize that the tadpole and froglets were raised in a common environment and that we tracked the same individual genotype across life stages.

### The effect of pool drying

While many studies have focused on how hydroperiod affects tadpole morphology and performance e.g. [39], [40], few have focused on how it affects froglet morphology and performance but see [7], [15]. In our study, exposure to decreasing water levels resulted in froglets with shorter femurs, tibias, and overall body lengths, all of which were also associated with reduced jump distance. It is generally believed that stressful environments adversely affect the performance of animals [41]. Our study and others [7], [15] clearly show that exposure to decreasing water levels produces froglets with low morphological trait values, and impaired jumping performance. However, shorter leg length and impaired jumping performance may not be a cost arising directly from the stress of the simulated pool drying treatment *per se*, but a consequence of the shorter development time induced by the treatment. Relyea [42] found that froglets emerging from containers with caged predators were no smaller than froglets from the control treatment, but they had a longer development time and relatively longer legs. This interpretation is further supported by a recent analysis of morphological differences within and between species of Spadefoot Toads and Parsley Frogs, where a longer larval period correlated with longer hind limbs, both within and across species [43]. The leg length of froglets thus seems to be a consequence of their development time and its hormonal regulation [43] and not a direct effect of the stressful environment *per se*. However other stressful conditions, such as high levels of competition for food, can result in longer development times being correlated with shorter leg length [36]. In contrast to the result found for froglets, which showed an impaired performance when reared in stressful environments, the stressful water level treatment produced tadpoles with better swimming performance than the treatment with a constant water level. Tadpoles subjected to the simulated pool drying treatment had shallower tails and bodies compared to those reared in the constant water level treatment. This change of body-shape in response to decreasing water levels has also been found in other studies of frogs [7], [44]. Interestingly, it was those tadpoles with the shallower body-shape induced by the stressful environment (decreasing water levels) that had the best swimming performance, which illustrates the decoupling of locomotor performance between the tadpole and froglet stages. The stressful environment thus induced a morphological change that produced tadpoles with a higher swimming speed and acceleration. Currently, we have no adaptive explanation for this, but it has previously been reported that animals that undergo accelerated development, usually have shallower bodies [7].

With regard to life history traits, the tadpoles responded to simulated pool drying by accelerating their development at the expense of a lower weight at metamorphosis. Under natural conditions the benefit of this response is obvious as it enables tadpoles to metamorphose before pools dry out [22]. However, one cost of a faster development time is the smaller size at metamorphosis, which has a negative impact on survival later on in life [25], [45]. Interestingly, tadpoles subjected to the constant water level treatment increased their weight by delaying metamorphosis, while no such relationship was found in tadpoles subjected to drying conditions, as has been observed in other amphibians [7]. When manipulating food levels, it is known that the positive relationship between development time and weight at metamorphosis is most pronounced under favourable conditions and becomes less important as growth conditions deteriorate [46], [47]. The finding that this relationship is weaker when development time is limited by pool drying, suggests that, under stressful conditions, organisms do not increase their size by delaying metamorphosis, whether the stress is caused by limited food or limited time.

### Correlations between morphology and locomotor performance

Though we did induce or measure antipredator performance in the presence of predators, our results on performance in terms of locomotion could be interpreted as an anti-predator performance trait. We emphasize that the body-shape of tadpoles had a large impact on their ability to escape simulated predator attacks: tadpoles with a shallow body and a relatively deep tail muscle exhibited improved swimming performance. This relationship corresponds to those found in other studies on other frog species [12], [26], [48], [49]. Dayton et al. [12] also found that, not only did a deep tail muscle increase swimming speed, but so did a deeper tail fin. In our study, however, increased tail fin depth did not translate into a better swimming performance. While the function of a deeper tail muscle seems to be clear, resulting in, for example, faster acceleration, the function of the tail fin depth is less clear (see discussion and references in Dayton et al. [12]).

All our morphological measurements on the froglets had a strong association with jumping ability. In contrast, size independent morphology (represented by PC2 score) exhibited a weak relationship with jumping length. Hence, as found in other studies, overall body size as well as leg-length, are usually good predictors of jumping performance in froglets [7], [15]. Since it has been suggested that jumping performance increases survival from attacking predators [50], [51] overall size, as well as relative leg length, should have important consequences for survival.

### Conclusions

We have shown that the locomotor performances that we measured were not correlated across life stages. Nevertheless some important size-dependent characters were not decoupled. For example, small size as a tadpole resulted in a short tibia length in froglets. Despite this, swimming performance and jump length were decoupled across life stages. The main reason for the absence of this correlation was that tadpole shape was not related to size. Metamorphosis can thus be seen as a way to decouple traits between life stages, although the performance of froglets remains somewhat constrained by size, which is determined in, and is carried over from, the tadpole stage. Although our study focused on a single performance trait in each developmental stage, there may be other performance traits that show different correlative patterns. However, we stress that, confronted with a risk of predation, the swimming performance of tadpoles and jump length of froglets are important traits that affect survival. Hence, our main conclusion is that while metamorphosis allows shifts and adaptation to very different habitats by decoupling traits across life stages, size-related traits are harder to decouple and may carry over fitness effects between different life stages.
